# Assessment of Trust in Physician: A Systematic Review of Measures

**DOI:** 10.1371/journal.pone.0106844

**Published:** 2014-09-10

**Authors:** Evamaria Müller, Jördis M. Zill, Jörg Dirmaier, Martin Härter, Isabelle Scholl

**Affiliations:** University Medical Center Hamburg-Eppendorf, Department of Medical Psychology, Hamburg, Germany; University of Pittsburgh Medical Center, United States of America

## Abstract

Over the last decades, trust in physician has gained in importance. Studies have shown that trust in physician is associated with positive health behaviors in patients. However, the validity of empirical findings fundamentally depends on the quality of the measures in use. Our aim was to provide an overview of trust in physician measures and to evaluate the methodological quality of the psychometric studies and the quality of psychometric properties of identified measures. We conducted an electronic search in three databases (Medline, EMBASE and PsycInfo). The secondary search strategy included reference and citation tracking of included full texts and consultation of experts in the field. Retrieved records were screened independently by two reviewers. Full texts that reported on testing of psychometric properties of trust in physician measures were included in the review. Study characteristics and psychometric properties were extracted. We evaluated the quality of design, methods and reporting of studies with the COnsensus based Standards for the selection of health status Measurement INstruments (COSMIN) checklist. The quality of psychometric properties was assessed with Terwee’s 2007 quality criteria. After screening 3284 records and assessing 169 full texts for eligibility, fourteen studies on seven trust in physician measures were included. Most of the studies were conducted in the USA and used English measures. All but one measure were generic. Sample sizes range from 25 to 1199 participants, recruited in very heterogeneous settings. Quality assessments revealed several flaws in the methodological quality of studies. COSMIN scores were mainly fair or poor. The overall quality of measures’ psychometric properties was intermediate. Several trust in physician measures have been developed over the last years, but further psychometric evaluation of these measures is strongly recommended. The methodological quality of psychometric property studies could be improved by adhering to quality criteria like the COSMIN checklist.

## Introduction

Patient-centeredness has gained importance in research, health policy and clinical practice. Trust is considered a central factor in determining a positive patient-physician relationship [1]–[3], which is an important dimension of patient-centeredness [4]. Trust in the context of healthcare has received increasing attention in the last two decades [5]. This is partly due to the voice of concerns about the effects of organizational changes in the healthcare system on patients’ trust in their healthcare professionals, healthcare institutions and the healthcare system itself [6], [7]. Patients’ trust has a particularly delicate notion, as patients who are ill and may have to face high risks regarding their health find themselves in an extremely vulnerable situation. Reliance on patients’ individual physicians and the healthcare system is often inevitable [6], [8]. The patient-physician relationship is characterized by a knowledge and power imbalance in which patients depend on the physicians’ expertise and execution of treatments to solve their health problems [6], [8], [9]. Hence, trust in physician plays an important role and has been studied extensively.

Trust in physician can be defined as the patient’s optimistic acceptance of a vulnerable situation and the belief that the physician will care for the patient’s interests [2]. Empirical studies have revealed that patients’ trust in physician is associated with patient satisfaction [10], continuity of care [11] and adherence to treatment [12]. Trust in physician facilitates access to healthcare, disclosure of relevant information and thereby supports accurate and timely diagnosis to be made [8]. Trust in physician is also associated with self-reported health improvement [13] and patients’ self-reported ability to manage their chronic disease [14]. As the body of work increases, the question of how to measure trust in physician gains importance. The validity of empirical findings is fundamentally dependent on the quality of the measures in use. Therefore, the selection of a measure should be carefully considered and based on the measure’s psychometric properties. Some studies addressed the quality of trust in physician measures [5], [7], [15], but no systematic review on trust in physician measures and their psychometric properties has been published to date. A thorough overview and comparison of different validated measures is needed a) to facilitate the choice of an appropriate instrument in accordance with the individual research purpose, b) to identify research gaps and needs for further psychometric testing of instruments and c) to inspire new measurement developments, if necessary.

Thus, the aims of this systematic review of measures on trust in the physician are 1) to identify existing psychometrically tested measures of trust in physician, 2) to determine the methodological quality of the studies that report on psychometric properties of measures, and 3) to evaluate the quality of identified measures based on their psychometric properties.

## Methods

### 2.1 Registration and search strategy

The protocol for this systematic review was registered in the International prospective register of systematic reviews PROSPERO [16] with the registration code CRD42013005048. We performed an electronic literature search using Medline, EMBASE and PsycInfo databases (via OVID). We identified relevant articles published between January 1979, the year of the first known measure of trust in physician [11] and the 21^st^ of June, 2013, when we administered the electronic literature search. For this purpose, we developed a detailed search strategy for each database (see Appendix S1). We considered a combination of the following four aspects appropriate: Trust AND the context of patient-physician interaction AND measurement AND psychometric properties. We adapted terms and keywords for each database and limited all searches to publications concerning adult, middle-aged or aged humans, published in either English or German. Full insight in the electronic database search strategy can be attained by consulting Appendix S1. Furthermore, we combined the electronic database search with a secondary search including reference and citation tracking of included full texts and consultation of experts in the field of research. Additionally, we screened references of a recently published review on trust in the health system [5].

### 2.2 Study selection

Two reviewers (EM and JZ) independently screened titles and abstracts of the identified records for possible inclusion in the study and independently assessed full texts for eligibility by applying exclusion criteria (see Table 1). We resolved differences concerning exclusion criteria by discussion until we reached consensus. If consensus could not be reached, the final decision was made by a third reviewer (IS).

**Table 1:** Exclusion criteria.

	*Exclusion criteria*	*Excluded full texts (n = 155)*
1	Publication is not inpeer-reviewed journal	5
2	Language of publicationother than English or German	2
3	Publication is not between 1979 and 2013	
4	Measured construct is nottrust (e.g. mistrust, distrust)	27
5	Trustee is not individual physician(e.g. dentist, nurse, health system, information)	14
6	Measure is not self-report questionnaire	4
7	Target group is not adult patients(e.g. children, parents, physicians, nurses)	1
8	Aim of study is not to testpsychometric properties of a scaleon trust in physician (e.g. subscale)	102
9	Not retrievable due to incomplete reference	
10	Full text not available	

Empty space = no full text was excluded for this reason.

### 2.3 Data extraction and quality assessments

We used data extraction sheets to collect study data and to make quality assessments. Data extraction sheets were pilot-tested and adjusted. Data extraction sheets comprised descriptive data of included studies and identified measures, and data on which quality assessments are based. We assessed the quality of design, methods and reporting of included studies on psychometric properties with the COnsensus-based Standards for the selection of health Measurement INstruments (COSMIN) checklist with a 4-point scale [17]–[19]. Furthermore, we evaluated the psychometric properties of identified measures with the quality criteria for good psychometric properties developed by Terwee et al. [20]. The quality criteria developed by Terwee [20] and the COSMIN checklist are described below. One reviewer (EM) performed data extraction and quality assessments. At the beginning of the quality rating, a double assessment of two studies was conducted by a second reviewer (IS) with whom ambiguities were discussed and resolved. The second reviewer (IS) further assisted with any questions occurring in the process of data extraction and quality evaluation.

#### 2.3.1 Quality of design, methods and reporting

The COSMIN checklist is based on an international Delphi study in which 57 experts found consensus on the definitions and assessments of measurement properties [17], [18]. The checklist rates the design, methodological and reporting quality of studies on measurement properties. There exist two versions for rating the COSMIN checklist: a dichotomous yes/no rating scale and a 4-point scale. The latter has been recommended to use in systematic reviews [19]. The COSMIN checklist comprises twelve boxes and assesses the following psychometric properties: A) internal consistency, B) reliability, C) measurement error, D) content validity, E) structural validity, F) hypotheses testing, G) cross-cultural validity, H) criterion validity, I) responsiveness and J) interpretability. For studies using item response theory methods, the IRT box provides evaluation. Sample data is extracted for each psychometric property separately with the generalizability box G. The IRT box and psychometric property boxes A to I can be evaluated with the 4-point scale. We performed data extraction and evaluation for the complete COSMIN checklist, but limit our presentation to the concise results of the 4-point scale ratings per psychometric property box. Item scores are excellent (+++), good (++), fair (+) or poor (0). The overall score for each box is determined by the lowest item score. Detailed information on the COSMIN checklist and the 4-point scale can be found on the COSMIN website [21].

#### 2.3.2 Quality of psychometric properties

The quality criteria for psychometric properties proposed by Terwee and colleagues [20] provide a condensed evaluation of measures’ psychometric properties and have been used in previous systematic reviews [22]. The Terwee criteria apply to the following properties: content validity, internal consistency, criterion validity, construct validity, reproducibility (agreement and reliability), responsiveness, floor and ceiling effects and interpretability. All properties are represented by one item that can be rated as positive (+), intermediate (?), negative (-) or no information available (0). We rated psychometric properties for each study separately, as they report on different study populations and results differ. For the exact definitions of psychometric properties and scoring criteria see the original publication [20].

## Results

### 3.1 Literature search and study selection

**Figure 1:**
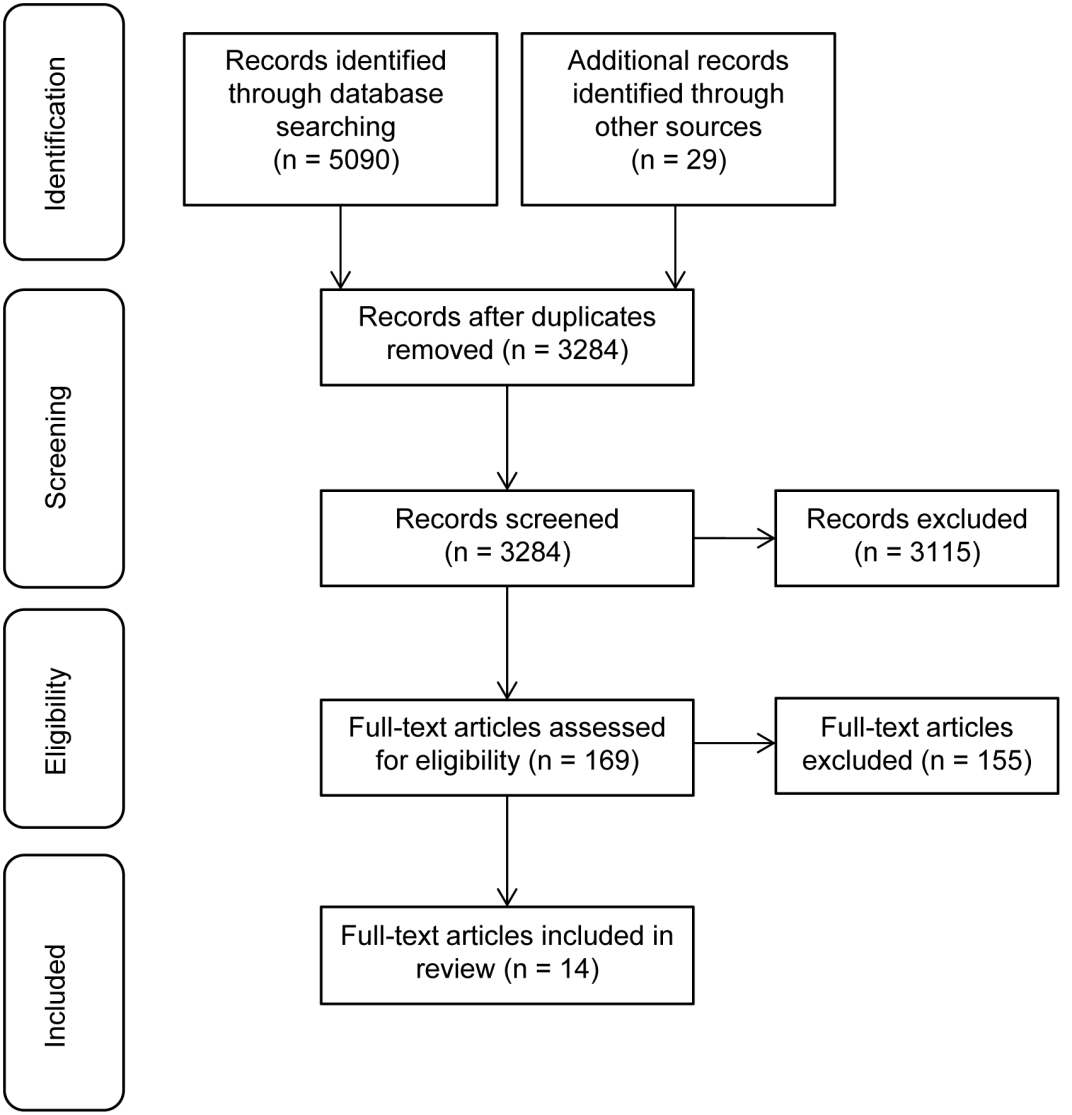
Flow diagram of study selection.

The electronic database search identified 5090 records. We found an additional number of 29 records through the secondary search. After removal of duplicates, the total search comprised 3284 records. We excluded 3115 records based on title- and abstract screening. Of the remaining 169 full texts, 155 full texts were excluded by applying exclusion criteria (see Table 1). The majority of full texts were excluded because the aim of the study was not to test psychometric properties of a scale on trust in physician. We included 14 studies in this review. The process of study selection is shown in Figure 1. We excluded some known measures of trust in physician such as the Kao scale [23] and the Safran scale [10]. They were excluded either because psychometric testing was not reported in peer-reviewed journal articles [23], [24] or trust in physician measures were subscales of instruments assessing a broader construct [10], [25]–[28].

### 3.2 Description of included studies and measures

Most of the studies were conducted in the USA and used English measures. Sample sizes range from 25 to 1199 participants. The majority of study samples included patients which were recruited in very heterogeneous settings. Most studies were based on outpatient samples [1], [11], [12], [29]–[34] with a variety of health issues. Included studies reported on psychometric properties of the following seven measures of trust in physician: the Trust in Physician Scale (TiPS), the Trust Scale for the Patient-Physician Dyad (TSPPD), the Wake Forest Physician Trust Scale (WFPTS) and a short form of the WFPTS, the Abbreviated Wake Forest Physician Trust Scale (A-WFPTS), the Health Care Relationship Trust Scale (HCRTS) and the further developed Health Care Relationship Trust Scale Revised (HCRTS-R), and the Trust in Oncologist Scale (TiOS). The TiOS, which was developed on the basis of the WFPTS, is the only population-specific measure and assesses cancer patients’ trust in their oncologists [35]. All measures are unidimensional and use a 5-point Likert response scale, except for the TSPPD. The TSPPD comprises two dimensions of benevolence and technical competence and can be rated on a 7-point Likert scale [33]. Descriptive data of included studies and identified measures are presented in Table 2.

**Table 2:** Descriptive data of trust in physician measures and included studies.

*Measure/Authors (Year)*	*Study sample*
**TiPS (Unidimensional, generic** * **, English, German, 11 items, 5-point Likert scale)**	
Anderson & Dedrick (1990)	Sample 1: non-insulin dependent diabetes mellitus outpatients
	(n = 160, 100% male, mean age 55.2 years, SD 10.5), USA
	Sample 2: non-insulin dependent diabetes mellitus outpatients
	(n = 106, 100% male, mean age 60.9 years, SD 9.5), USA
Thom et al. (1999)	Primary care patients
	(n = 414, 62% female, mean age 47.3 years, SD 16.2), USA
Freburger et al. (2003)	Rheumatoid arthritis, osteoarthritis, fibromyalgia outpatients
	(n = 713, 77% female, mean age 59.6 years, SD 12.7), USA
Glattacker et al. (2007)	Patients of orthopaedic rehabilitation centres
	(n = 460, 60% female, mean age 68.4 years, SD 8.2), Germany
Krajewska-Kulak et al. (2011)	Hospitalized patients of obstetrics and gynaecology departments
	(n = 259, 100% female, mean age 56.2 years, SD 3.5), Poland, Greece
**TSPPD (Bidimensional, generic** * **, English, 51 items, 7-point Likert scale)**	
Leisen & Hyman (2001)	Sample 1: Undergraduate students of research university
	(n = 161, 62% male, mean age 23 years), USA
	Sample 2: Employees of service organization covered by managed care plan
	(n = 214, 34.1% male, mean age 45.6 years), USA
**WFPTS (Unidimensional, generic** * **, English, Dutch, 10 items, 5-point Likert scale)**	
Hall et al. (2002)	Sample 1: health-insured US citizens with physician contact in the last 2 years
	(n = 959, 67.8% female, mean age 48.8 years, SD 17.2), USA
	Sample 2: health maintenance organization members
	(n = 1199, 55.5% female, mean age 46.5 years, SD 11.2), USA
Bachinger et al. (2008)	Outpatients of internal medical clinic
	(n = 201, 59.7% female, mean age 50.0 years, SD 14.8), Netherlands
Donnelly et al. (2011)	Hospitalized patients of forensic hospital
	(n = 81, 92.6% male, mean age 46.6 years, SD 12.4), Ireland
**A-WFPTS (Unidimensional, generic** * **, English, 5 items, 5-point Likert scale)**	
Dugan et al. (2005)	Sample 1: health-insured US citizens with physician contact in the last 2 years
	(n = 1064, 68% female, mean age 49.8 years), USA
	Sample 2: health maintenance organization members
	(n = 1045, 55% female, mean age 46.6 years), USA
**HCRTS (Unidimensional, generic** * **, English, 15 items, 5-point Likert scale)**	
Bova et al. (2006)	Sample 1: HIV-infected outpatients
	(n = 25, 72% female, mean age 41.5 years, SD 6.1), USA
	Sample 2: HIV-infected outpatients
	(n = 99, 49.5% female, mean age 42.9 years, SD 7.8), USA
**HCRTS-R (Unidimensional generic** * **, English, 13 items, 5-point Likert scale)**	
Bova et al. (2012)	Primary care patients
	(n = 431, 60.1% female, mean age 55.6 years, SD 16.1), USA
**TiOS (Unidimensional, population-specific, English, Dutch, 18 items, 5-point Likert scale)**	
Hillen et al. (2012)	Cancer patients
	(n = 423, 57% male, median age 63 years, range 19–90), Netherlands
Hillen et al. (2013)	Cancer patients
	(n = 175, 43% female, median age 62 years, range 21–88), Australia

Bold lines show descriptive data of measures. SD = standard deviation.

*With “generic measures”, we mean measures that are applicable to a broad range of medical conditions and in different specialties.

### 3.3 Quality of design, methods and reporting

Assessment of the quality of design, methods and reporting of psychometric property studies with the COSMIN checklist are shown in Table 3. All included studies reported on internal consistency (Box A) and COSMIN rating could be applied. Studies on the TiPs received three poor [29], [34], [36], one fair [37] and one good [32] score for internal consistency. The study on the TSPPD [33] received a poor score. The WFPTS shows mixed results with one good study rating [1] and two fair ratings [11], [38] for internal consistency. The internal consistency scores for studies on A-WFPTS [12], HCRTS [30] and HCRTS-R [31] were good. Studies on the TiOS received one good [35] and one fair [39] rating for internal consistency. Few studies assessed reliability (Box B) and rating could be applied to five studies. Scores were either fair or poor. Studies reporting on the reliability of the TiPS [34] and the TiOS [35] were rated as fair. Studies assessing reliability of the WFPTS [11], [38] and the HCRTS [30] received poor scores. None of the studies reported on the psychometric property measurement error (Box C). Ratings for content validity (Box D) were made for studies reporting on the initial development of measures. Scores were good for the TiPS [29], WFPTS [11], HCRTS [30] and TiOS [35], but the study on the TSPPD [33] received a poor score for content validity. Structural validity (Box E) was assessed by most studies and the major part scored fair or good. Structural validity assessments of the TiPS [32], [37] were rated as fair, whereas the study on the TSPPD [33] scored poorly. Results for studies on the WFPTS and TiOS were mixed for structural validity. Studies on the WFPTS scored good [1] and fair [11], [38]. Reports on the structural validity of the TiOS were rated as good [35] and fair [39]. Structural validity ratings were good for studies reporting on the A-WFPTS [12], HCRTS [30] and HCRTS-R [31]. Hypotheses testing rating (Box F) applied to all studies. Results were either fair or poor. One study on the TiPS [32] and WFPTS [1] each, as well as the studies reporting on the A-WFPTS [12] and HCRTS-R [31] scored fair. Cross-cultural validity (Box G) was assessed by four studies. Rating applied to studies on the TiPS [36], [37], WFPTS [1] and TiOS [39]. All studies received poor ratings for cross-cultural validity. The measurement properties criterion validity (Box H) and responsiveness (Box I) were not assessed by any of the studies. Detailed results for COSMIN ratings on item level are shown in Appendix S2.

**Table 3:** Quality of design, methods and reporting of studies on psychometric properties.

*Measure*	*Authors (Year)*	*Psychometric properties*								
		A	B	C	D	E	F	G	H	I
**TiPS**	Anderson & Dedrick (1990)	0			++		0			
	Thom et al. (1999)	0	+				0			
	Freburger et al. (2003)	++				+	+			
	Glattacker et al. (2007)	+				+	0	0		
	Krajewska-Kulak et al. (2011)	0					0	0		
**TSPPD**	Leisen & Hyman (2001)	0			0	0	0			
**WFPTS**	Hall et al. (2002)	+	0		++	+	0			
	Bachinger et al. (2009)	++				++	+	0		
	Donnelly et al. (2011)	+	0			+	0			
**A-WFPTS**	Dugan et al. (2005)	++				++	+			
**HCRTS**	Bova et al. (2006)	++	0		++	++	0			
**HCRTS-R**	Bova et al. (2012)	++				++	+			
**TiOS**	Hillen et al. (2012)	++	+		++	++	0			
	Hillen et al. (2013)	+				+	0	0		

COSMIN psychometric property boxes: A = internal consistency, B = reliability, C = measurement error, D = content validity, E = structural validity, F = hypotheses testing, G = cross-cultural validity, H = criterion validity, I = responsiveness. 4-point scale rating: +++ = excellent, ++ = good, + = fair, 0 = poor, empty space = COSMIN rating not applicable. For exact information regarding the definitions of psychometric properties and 4-point scale rating see COSMIN website [21].

### 3.4 Quality of psychometric properties

Quality ratings of measures’ psychometric properties assessed with the Terwee criteria are presented in Table 4. Studies reporting on the initial development of measures [11], [29], [30], [35] received positive scores for content validity, except for the study reporting on the development of the TSPPD [33]. Scores for internal consistency were all positive for studies on the WFPTS [1], [11], [38], the A-WFPTS [12], and the TiOS [35], [39]. Studies on the TiPS received positive [32], [37] and intermediate [29], [34], [36] scores. The TSPPD [33] and the HCRTS [30] scored intermediately. The HCRTS-R [31] received the only negative score for internal consistency. Criterion validity was not assessed by any of the studies. Construct validity was mainly rated as intermediate [12], [30], [31], [33]. The TiPS received one positive [37] and three intermediate ratings [29], [32], [34]. Similarly, the WFPTS scored intermediately twice [11], [38] and positive once [1]. Construct validity scores of the TiOS were mixed with a positive [35] and negative [39] rating each. Few studies provided data on the measurement property reproducibility. The reproducibility aspect agreement was not assessed by any of the studies, whereas some studies present data on the reproducibility aspect reliability. The single study that assessed reliability for the TiPS [34] scored positively. Reliability of the WFPTS [11], [38], HCRTS [30] and TiOS [35] was rated as intermediate. The measurement property responsiveness was not assessed by any of the studies. Floor and ceiling effects were assessed for the TiPS, A-WFPTS, HCRTS and HCRTS-R. The English version of the TiPS [32], [34] scored positively, but the German version [37] received a negative score for floor and ceiling effects. The A-WFPTS [12] scored intermediately. The HCRTS [30] and HCRTS-R [31] received negative scores for floor and ceiling effects. Ratings for interpretability were all intermediate and available for the TiPS [32], [34], [37], WFPTS [1], [11], [38], A-WFPTS [12], HCRTS-R [31] and TiOS [35], [39].

**Table 4:** Quality of psychometric properties.

*Instruments/Authors (Year)*	*Content validity*	*Internal consistency*	*Criterion validity*	*Construct* *validity*	*Reproducibility* *(Agreement)*	*Reproducibility* *(Reliability)*	*Responsiveness*	*Floor & ceiling effects*	*Interpretability*
**TiPS**	**+0000**	**??++?**	**00000**	**???+0**	**00000**	**0+000**	**00000**	**0++–0**	**0???0**
Anderson & Dedrick (1990)	+	?	0	?	0	0	0	0	0
Thom et al. (1999)	0	?	0	?	0	+	0	+	?
Freburger et al. (2003)	0	+	0	?	0	0	0	+	?
Glattacker et al. (2007)	0	+	0	+	0	0	0	–	?
Krajewska-Kulak et al. (2011)	0	?	0	0	0	0	0	0	0
**TSPPD**	**?**	**?**	**0**	**?**	**0**	**0**	**0**	**0**	**0**
Leisen & Hyman (2001)	?	?	0	?	0	0	0	0	0
**WFPTS**	**+00**	**+++**	**000**	**?+?**	**000**	**?0?**	**000**	**000**	**???**
Hall et al. (2002)	+	+	0	?	0	?	0	0	?
Bachinger et al. (2009)	0	+	0	+	0	0	0	0	?
Donnelly et al. (2011)	0	+	0	?	0	?	0	0	?
**A-WFPTS**	**0**	**+**	**0**	**?**	**0**	**0**	**0**	**?**	**?**
Dugan et al. (2005)	0	+	0	?	0	0	0	?	?
**HCRTS**	**+**	**?**	**0**	**?**	**0**	**?**	**0**	**–**	**0**
Bova et al. (2006)	+	?	0	?	0	?	0	–	0
**HCRTS-R**	**0**	**–**	**0**	**?**	**0**	**0**	**0**	**–**	**?**
Bova et al. (2012)	0	–	0	?	0	0	0	–	?
**TiOS**	**+0**	**++**	**00**	**+–**	**00**	**?0**	**00**	**00**	**??**
Hillen et al. (2012)	+	+	0	+	0	?	0	0	?
Hillen et al. (2013)	0	+	0	–	0	0	0	0	?

Rating: + = positive, ?  = intermediate, − = negative, 0 = no information available. Bold lines summarize ratings of psychometric properties per measure. For exact information regarding the definitions of psychometric properties see Terwee et al [20].

## Discussion

This systematic review included fourteen studies on seven measures of trust in physician. Most studies were conducted in the USA and reported on psychometric properties of the TiPS or the WFPTS and its abbreviated version. Samples varied enormously in size and participants’ characteristics. Quality assessments with the COSMIN checklist and the Terwee criteria revealed a heterogeneous picture of the methodological quality of included studies and the quality of psychometric properties of identified measures.

Regarding the results of the COSMIN rating for the design, methods and reporting of psychometric studies, several research gaps became apparent. With a total of five different studies [29], [32], [34], [36], [37], the TiPS is the measure which has been most extensively tested. However, the majority of studies on the TiPS were rated poor for internal consistency [29], [34], [36]. Only two of the studies on the TiPS assessed structural validity [32], [37], and the quality of these assessments was rated as fair. COSMIN results for all psychometric studies reveal that only a selection of psychometric properties was reported and ratings were mainly fair or poor. Internal consistency and hypotheses testing were addressed in all of the studies, but quality ratings with the COSMIN checklist revealed serious flaws in more than 70% of the studies’ reports on this psychometric property [11], [29], [30], [33]–[39]. Few studies assessed reliability [11], [30], [34], [35], [38] or cross-cultural validity [1], [36], [37], [39], and the quality of these assessments was rated as poor, except for two studies with fair reporting [34], [35]. The psychometric properties measurement error, criterion validity and responsiveness were not addressed in any of the studies. Looking at the COSMIN ratings per study, two studies received poor scores for all reported psychometric properties. These studies are the measure development study of the TSPPD [33] and a cross-cultural validation study of the TiPS [36]. The measure development study of the TiOS [35] had the best quality regarding the design, methods and reporting of psychometric property assessment, closely followed by the study on the HCRTS [30].

Remarkably, none of the studies scored excellent on any psychometric property in the COSMIN evaluation. Looking at the results of COSMIN items (see Appendix B), studies scored excellent in many respects. Yet, this is not reflected in COSMIN scores for psychometric properties. The “worst score counts” policy of COSMIN leads to a negatively biased view on the studies’ design, methods and reporting. However, as all items represent aspects considered very important by the COSMIN Delphi panel, poor ratings for any of the items should be considered as serious flaws [19]. Overall, the results of this review show that the methodological quality of psychometric property studies on trust in physician is not satisfactory in many respects. However, the more recently published measure development studies [30], [35] better met with the COSMIN criteria and had reasonably good results for most reported psychometric properties.

To give an overview of the quality of psychometric properties assessed with the Terwee criteria, we composed a table (see Table 4) with quality ratings presented for each study individually. Overall, the quality of psychometric properties of trust in physician measures was intermediate. For some measures, psychometric properties were assessed in a variety of study populations and quality judgments per measure differ. For example, the TiPS had positive ratings for floor and ceiling effects in two studies of the English version [32], [34], whereas floor and ceiling effects of the German version [37] were judged negatively. Content validity ratings were positive for all measure development studies [11], [29], [30], [35], but for the development study of the TSPPD [33]. The use of a measure is only recommended, if content validity is adequate [20]. Looking at the quality judgments of measures per study, the TSPPD [33] had the worst quality. Consequently, the TSPPD would not be recommended to use without further psychometric evaluation. The measure development study of the TiOS [35] received the best quality ratings for psychometric properties.

However, our results concerning the quality of psychometric properties evaluated with the Terwee criteria need to be considered carefully. The assessment of the methodological quality of studies with the COSMIN checklist indicated that many studies lack quality of design, methods and reporting. Judgment on the quality of a measure can only be as good as the basis for evaluation [20]. In this review, the basis for evaluation is the studies’ reports of psychometric property assessments and outcomes. Hence, some of the measures evaluated here, may have received worse quality judgments for psychometric properties due to flaws in the study’s reporting. Viewing the quality of psychometric properties in the light of the studies’ quality of design, methods and reporting, the TiOS is the measure with the best psychometric properties evaluated in the methodologically best study.

The results of this review can be used to assist researchers in choosing a measure optimal for their individual research purpose. However, it is important to note that a measure’s psychometric properties need to be re-established for any new setting, sample or cultural context [40].

The present systematic review has several positive qualities: First, we used a complex and detailed search strategy in the electronic database search to retrieve all records relevant to our purpose. Second, two reviewers independently assessed records and full texts for possible inclusion in the study. Third, we performed two quality assessments by using both the COSMIN checklist with 4-point scale rating and the quality criteria for good psychometric properties developed by Terwee et al. [20]. This combination has been recommended to use for the separate evaluation of the methodological quality of studies and the quality of their results [17]. Judgment on the quality of studies provides the background for the interpretation of psychometric properties reported in the studies. Thus, a strength of this review is that it supplies both, a condensed evaluation of the quality of studies and of their results. This review has several limitations: First, our search was limited to studies published from 1979 onwards, limited to English and German, and we searched only three databases. As a consequence, we might have missed relevant publications. However, we carried out a thorough secondary search to limit this possibility to a minimum. Second, data extraction and quality evaluation of included studies was performed by one reviewer only. This may have led to a biased assessment of included studies and measures’ psychometric properties. However, we performed a double assessment of two studies in the beginning of the quality assessments and discussed any ambiguities occurring in the process of quality assessments to reduce this bias. Furthermore, as every systematic review, our results are limited by our inclusion and exclusion criteria and we might have missed certain interesting scales, e.g. a paper on the Spanish version of the WFPTS that did not aim to test psychometric properties [41] and a paper on a measure that assesses trust in physicians in general [42].

In this review, we identified seven psychometrically evaluated measures of trust in physician. These measures cover a multitude of research needs, as they are mainly generic and include short as well as long scales validated in diverse study populations. Hence, the development of new measures does not seem necessary. However, the mixed results of the Terwee quality criteria for psychometric properties in different studies indicate that further psychometric evaluation is strongly recommended. The quality assessment of psychometric studies with the COSMIN checklist revealed several research gaps. Content areas like measurement error, criterion validity and responsiveness have been neglected in the studies to date and should be addressed in future psychometric studies. The results of the COSMIN checklist for hypotheses testing indicate serious flaws in the methodological quality of present evaluation studies. Hence, hypotheses testing should receive special attention in future psychometric evaluation studies. Cross-cultural validity was addressed in only four studies [1], [36], [37], [39] and the methodological quality of these studies was rated as poor. However, translations of measures are needed to support research on trust in physician worldwide. The applicability of translated measures should be assessed in cross-cultural validity studies for different languages and cultural contexts [43]. Moreover, investigation of psychometric properties should adhere to standards for assessing psychometric properties like the COSMIN checklist in order to contribute to the quality of future studies and facilitate the comparison of their results.

In conclusion, this systematic review identified several trust in physician measures and serious gaps in the psychometric property evaluation of some of these measures. Good quality measures are needed to assess trust in physician in empirical studies in the context of healthcare.

## Supporting Information

Appendix S1 **Electronic database search strategy for Medline, EMBASE, PsycInfo.**
(DOCX)

Appendix S2 **Detailed results for the COSMIN checklist with 4-point scale rating.** *Description of item content altered to fit this table. For exact item content see COSMIN website (www.cosmin.nl). Study IDs: T1 = Anderson & Dedrick (1990), T2 = Thom et al. (1999), T3 = Freburger et al. (2003), T4 = Glattacker et al. (2007), T5 = Krajewska-Kulak et al. (2011), T6 = Leisen & Hyman (2001), T7 = Hall et al. (2002), T8 = Bachinger et al. (2008), T9 = Donnelly et al. (2011), T10 = Dugan et al. (2005), T11 = Bova et al. (2006), T12 = Bova et al. (2012), T13 = Hillen et al. (2012), T14 = Hillen et al. (2013). 4-point scale rating: +++ = excellent, ++ = good, + = fair, 0 = poor, empty space = COSMIN rating not applicable. n/a = not applicable.
(DOCX)

Checklist S1 **PRISMA checklist.**
(DOC)

## References

[1] Bachinger SM, Kolk AM, Smets EM (2009) Patients’ trust in their physician - Psychometric properties of the Dutch version of the “Wake Forest Physician Trust Scale”. Patient Educ Couns 76: 126–131. 19136228 10.1016/j.pec.2008.11.020

[2] Hall MA, Dugan E, Zheng B, Mishra AK (2001) Trust in physicians and medical institutions: What is it, can it be measured, and does it matter? Milbank Q 79: 613–639. 11789119 10.1111/1468-0009.00223 PMC2751209

[3] Ommen O, Janssen C, Neugebauer E, Bouillon B, Rehm K, et al (2008) Trust, social support and patient type - Associations between patients perceived trust, supportive communication and patients preferences in regard to paternalism, clarification and participation of severely injured patients. Patient Educ Couns 73: 196–204. 18450408 10.1016/j.pec.2008.03.016

[4] Scholl I, Zill JM, Härter M, Dirmaier J (2012) Dimensions of patient-centeredness - A conceptual view; University of St Andrews, Scotland, UK.

[5] Ozawa S, Sripad P (2013) How do you measure trust in the health system? A systematic review of the literature. Soc Sci Med 91: 10–14. 23849233 10.1016/j.socscimed.2013.05.005

[6] Hillen MA, de Haes HC, Smets EM (2011) Cancer patients’ trust in their physician - A review. Psycho-oncology 20: 227–241. 20878840 10.1002/pon.1745

[7] Pearson SD, Raeke LH (2000) Patients’ trust in physicians: Many theories, few measures, and little data. J Gen Intern Med 15: 509–513. 10940139 10.1046/j.1525-1497.2000.11002.x PMC1495476

[8] Calnan M, Rowe R (2006) Researching trust relations in health care: Conceptual and methodological challenges - An introduction. J Health Organ Manag 20: 349–358. 17087399 10.1108/14777260610701759

[9] Becker ER, Roblin DW (2008) Translating primary care practice climate into patient activation: The role of patient trust in physician. Med Care 46: 795–805. 18665059 10.1097/MLR.0b013e31817919c0

[10] Safran DG, Kosinski M, Tarlov AR, Rogers WH, Taira DA, et al (1998) The primary care assessment survey: Tests of data quality and measurement performance. Med Care 36: 728–739. 9596063 10.1097/00005650-199805000-00012

[11] Hall MA, Zheng B, Dugan E, Camacho F, Kidd KE, et al (2002) Measuring patients’ trust in their primary care providers. Med Care Res Rev 59: 293–318. 12205830 10.1177/1077558702059003004

[12] Dugan E, Trachtenberg F, Hall MA (2005) Development of abbreviated measures to assess patient trust in a physician, a health insurer, and the medical profession. BMC Health Serv Res 5: 64. 16202125 10.1186/1472-6963-5-64 PMC1262715

[13] Safran DG, Taira DA, Rogers WH, Kosinski M, Ware JE, et al (1998) Linking primary care performance to outcomes of care. J Fam Pract 47: 213–220. 9752374

[14] Bonds DE, Camacho F, Bell RA, Duren-Winfield VT, Anderson RT, et al (2004) The association of patient trust and self-care among patients with diabetes mellitus. BMC Fam Pract 5: 26. 15546482 10.1186/1471-2296-5-26 PMC535564

[15] Hall MA (2006) Researching medical trust in the United States. J Health Organ Manag 20: 456–467. 17087405 10.1108/14777260610701812

[16] Mavis B, Turner J, Lovell K, Wagner D (2006) Faculty, students, and actors as standardized patients: Expanding opportunities for performance assessment. Teach Learn Med 18: 130–136. 16626271 10.1207/s15328015tlm1802_7

[17] Mokkink LB, Terwee CB, Patrick DL, Alonso J, Stratford PW, et al (2010) The COSMIN checklist for assessing the methodological quality of studies on measurement properties of health status measurement instruments: An international Delphi study. Qual Life Res 19: 539–549. 20169472 10.1007/s11136-010-9606-8 PMC2852520

[18] Mokkink LB, Terwee CB, Patrick DL, Alonso J, Stratford PW, et al (2010) The COSMIN study reached international consensus on taxonomy, terminology, and definitions of measurement properties for health-related patient-reported outcomes. J Clin Epidemiol 63: 737–745. 20494804 10.1016/j.jclinepi.2010.02.006

[19] Terwee CB, Mokkink LB, Knol DL, Ostelo RWJG, Bouter LM, et al (2012) Rating the methodological quality in systematic reviews of studies on measurement properties: A scoring system for the COSMIN checklist. Qual Life Res 21: 651–657. 21732199 10.1007/s11136-011-9960-1 PMC3323819

[20] Terwee CB, Bot SD, de Boer MR, van der Windt DA, Knol DL, et al (2007) Quality criteria were proposed for measurement properties of health status questionnaires. J Clin Epidemiol 60: 34–42. 17161752 10.1016/j.jclinepi.2006.03.012

[21] COSMIN: Consensus-based Standards for the selection of health Measurement INstruments, www.cosmin.nl, 2013 June 23.

[22] Tijssen M, van Cingel R, van Melick N, de Visser E (2011) Patient-reported outcome questionnaires for hip arthroscopy: A systematic review of the psychometric evidence. BMC Musculoskelet Disord 12: 117. 21619610 10.1186/1471-2474-12-117 PMC3129322

[23] Kao A, Green DC, Zaslavski A, Koplan JP, Cleary PD (1998) The relationship between method of physician payment and patient trust. JAMA 280: 1709–1714. 10.1001/jama.280.19.1708 9832007

[24] Caterinicchio RP (1979) Testing plausible path models of interpersonal trust in patient-physician treatment relationships. Soc Sci Med Med Psychol Med Sociol 13A: 81–99. 551529 10.1016/0160-7979(79)90011-0

[25] Egede LE, Ellis C (2008) Development and testing of the multidimensional trust in health care systems scale. J Gen Intern Med 23: 808–815. 18415653 10.1007/s11606-008-0613-1 PMC2517872

[26] Fondacaro M, Frogner B, Moos R (2005) Justice in health care decision-making: Patients’ appraisals of health care providers and health plan representatives. Soc Justice Res 18: 63–81. 16021741 10.1007/s11211-005-3393-3 PMC2878657

[27] Ramsay J, Campbell JL, Schroter S, Green J, Roland M (2000) The General Practice Assessment Survey (GPAS): Tests of data quality and measurement properties. Fam Pract 17: 372–379. 11021894 10.1093/fampra/17.5.372

[28] Weech-Maldonado R, Carle A, Weidmer B, Hurtado M, Ngo-Metzger Q, et al (2012) The consumer assessment of healthcare providers and systems (CAHPS) cultural competence (CC) item set. Med Care 50: S22–S31. 22895226 10.1097/MLR.0b013e318263134b PMC3748811

[29] Anderson LA, Dedrick RF (1990) Development of the Trust in Physician scale: A measure to assess interpersonal trust in patient-physician relationships. Psychol Rep 67: 1091–1100. 2084735 10.2466/pr0.1990.67.3f.1091

[30] Bova C, Fennie KP, Watrous E, Dieckhaus K, Williams AB (2006) The Health Care Relationship (HCR) Trust Scale: Development and psychometric evaluation. Res Nurs Health 29: 477–488. 16977644 10.1002/nur.20158

[31] Bova C, Route PS, Fennie K, Ettinger W, Manchester GW, et al (2012) Measuring patient-provider trust in a primary care population: Refinement of the health care relationship trust scale. Res Nurs Health 35: 397–408. 22511461 10.1002/nur.21484

[32] Freburger JK, Callahan LF, Currey SS, Anderson LA (2003) Use of the Trust in Physician Scale in patients with rheumatic disease: Psychometric properties and correlates of trust in the rheumatologist. Arthritis Care Res 49: 51–58. 10.1002/art.10925 12579593

[33] Leisen B, Hyman MR (2001) An improved scale for assessing patients’ trust in their physician. Health Mark Q 19: 23–42. 10.1300/J026v19n01_03 11727290

[34] Thom DH, Ribisl KM, Stewart AL, Luke DA (1999) Further validation and reliability testing of the Trust in Physician Scale. Med Care 37: 510–517. 10335753 10.1097/00005650-199905000-00010

[35] Hillen MA, Koning CCE, Wilmink JW, Klinkenbijl JHG, Eddes EH, et al (2012) Assessing cancer patients’ trust in their oncologist: Development and validation of the Trust in Oncologist Scale (TiOS). Support Care Cancer 20: 1787–1795. 21947560 10.1007/s00520-011-1276-8 PMC3390706

[36] Krajewska-Kulak E, Chilicka M, Kulak W, Adraniotis J, Chadzopulu A, et al (2011) Assessment of physician-patient trust in the obstetrics and gynecology departments in Poland and Greece. Ginekol Pol 82: 905–910. 22384626

[37] Glattacker M, Guelich M, Farin E, Jaeckel WH (2007) Trust in the physician - Psychometric testing of the German version of the “Trust in physician scale”. Phys Med Rehab Kuror 17: 141–148.

[38] Donnelly V, Lynch A, Devlin C, Naughton L, Gibbons O, et al (2011) Therapeutic alliance in forensic mental health: Coercion, consent and recovery. Ir J Psychol Med 28: 21–28. 10.1017/S0790966700011861 30199989

[39] Hillen MA, Butow PN, Tattersall MH, Hruby G, Boyle FM, et al (2013) Validation of the English version of the Trust in Oncologist Scale (TiOS). Patient Educ Couns 91: 25–28. 23219483 10.1016/j.pec.2012.11.004

[40] Streiner DL, Norman GR (2008) Health measurement scales - A practical guide to their development and use. Oxford: Oxford University Press.

[41] Vissman AT, Young AM, Wilkin AM, Rhodes SD (2013) Correlates of HAART adherence among immigrant Latinos in the southeastern United States. AIDS Care 25: 356–363. 22835082 10.1080/09540121.2012.701722

[42] Hall MA, Camacho F, Dugan E, Balkrishnan R (2002) Trust in the medical profession: Conceptual and measurement issues. Health Serv Res 37: 1419–1439. 12479504 10.1111/1475-6773.01070 PMC1464022

[43] Sperber AD (2004) Translation and validation of study instruments for cross-cultural research. Gastroenterology 126: S124–S128. 14978648 10.1053/j.gastro.2003.10.016

