# Supplementary material for: Assessment of Trust in Physician: A Systematic Review of Measures
Source: PLoS One. 2014 Sep 10;9(9):e106844. doi: 10.1371/journal.pone.0106844 (PMC4160203; doi:10.1371/journal.pone.0106844)
Supplement: Appendix S1 — Electronic database search strategy for Medline, EMBASE, PsycInfo. (DOCX) [file pone.0106844.s001.docx]

# Appendix S2: Electronic database search strategy for Medline, EMBASE, PsycInfo

**Medline database via Ovid**

Trust* (mp) AND

exp Physician-Patient Relations*(OR exp Patients/psychology*(pt)/ Physician* (mp)/ Patient* (mp)/ Doctor* (mp)/ Clinician* (mp)/ General practic* (mp)) AND

exp Questionnaires (OR exp Outcome Assessment (Health Care)/ Instrument* (mp)/ Measur* (mp)/ Index (mp)/ Indices (mp)/ Scale* (mp)/ Questionnair* (mp)/ Self report* (mp)/ Health survey* (mp)/ Test* (mp)) AND

exp Psychometrics (OR exp factor analysis, statistical/ exp ROC Curve/ exp “Sensitivity and Specificity”/ exp “Reproducibility of Results”/ Reliability (mp)/ Reliabl* (mp)/ Pychometr* (mp)/ Factor* (mp)/ Valid* (mp)/ Analys* (mp)/ Analyz* (mp)/ Factor structure (mp)/ Factor analysis (mp)/ Face validity (mp)/ Criterion validity (mp)/ Convergent validity (mp)/ Internal consistency (mp)/ Test-retest (mp)/ Cronbach* (mp)/ Rasch analysis (mp)/ Item response model (mp)/ Item response theory (mp)/ Differential item function (mp)/ Cross-cultural validation (mp)/ Item reduction (mp)/ Kappa (mp)/ Responsiveness (mp)/ Intraclass correlat* (mp))

**EMBASE database via Ovid**

Trust* (mp) AND

exp doctor patient relation (OR exp physician/ exp patient/ Physician* (mp)/ Patient* (mp)/ Doctor* (mp)/ Clinician* (mp)/ General practic* (mp)) AND

exp questionnaire (OR exp health survey/ exp outcome assessment/ exp rating scale/ Instrument* (mp)/ Measur* (mp)/ Index (mp)/ Indices (mp)/ Scale* (mp)/ Questionnair* (mp)/ Self report* (mp)/ Health survey* (mp)/ Test* (mp)) AND

exp psychometry (OR exp factorial analysis/ Reliability (mp)/ Reliabl* (mp)/ Pychometr* (mp)/ Factor* (mp)/ Valid* (mp)/ Analys* (mp)/ Analyz* (mp)/ Factor structure (mp)/ Factor analysis (mp)/ Face validity (mp)/ Criterion validity (mp)/ Convergent validity (mp)/ Internal consistency (mp)/ Test-retest (mp)/ Cronbach* (mp)/ Rasch analysis (mp)/ Item response model (mp)/ Item response theory (mp)/ Differential item function (mp)/ Cross-cultural validation (mp)/ Item reduction (mp)/ Kappa (mp)/ Responsiveness (mp)/ Intraclass correlat* (mp))

**PsycInfo database via Ovid**

Trust* (mp) AND

exp physicians (OR exp patients/ exp clinicians/ exp general practitioners/ Physician* (mp)/ Patient* (mp)/ Doctor* (mp)/ Clinician* (mp)/ General practic* (mp)) AND

exp measurement (OR exp questionnaires/ Instrument* (mp)/ Measur* (mp)/ Index (mp)/ Indices (mp)/ Scale* (mp)/ Questionnair* (mp)/ Self report* (mp)/ Health survey* (mp)/ Test* (mp)) AND

exp psychometrics (OR exp factor analysis/ exp factor structure/ exp test validity/ exp statistical validity/ exp statistical reliability/ exp test reliability/ exp interrater reliability/ Reliability (mp)/ Reliabl* (mp)/ Pychometr* (mp)/ Factor* (mp)/ Valid* (mp)/ Analys* (mp)/ Analyz* (mp)/ Factor structure (mp)/ Factor analysis (mp)/ Face validity (mp)/ Criterion validity (mp)/ Convergent validity (mp)/ Internal consistency (mp)/ Test-retest (mp)/ Cronbach* (mp)/ Rasch analysis (mp)/ Item response model (mp)/ Item response theory (mp)/ Differential item function (mp)/ Cross-cultural validation (mp)/ Item reduction (mp)/ Kappa (mp)/ Responsiveness (mp)/ Intraclass correlat* (mp))

All searches limited to

• human

• 1979-current

• adults, middle aged and aged

• English and German

Electronic database searches were conducted on the 21^st^ of June in 2013.
