# Supplementary material for: Assessment of Trust in Physician: A Systematic Review of Measures
Source: PLoS One. 2014 Sep 10;9(9):e106844. doi: 10.1371/journal.pone.0106844 (PMC4160203; doi:10.1371/journal.pone.0106844)
Supplement: Appendix S2 — Detailed results for the COSMIN checklist with 4-point scale rating. *Description of item content altered to fit this table. For exact item content see COSMIN website (www.cosmin.nl). Study IDs: T1 = Anderson & Dedrick (1990), T2 = Thom et al. (1999), T3 = Freburger et al. (2003), T4 = Glattacker et al. (2007), T5 = Krajewska-Kulak et al. (2011), T6 = Leisen & Hyman (2001), T7 = Hall et al. (2002), T8 = Bachinger et al. (2008), T9 = Donnelly et al. (2011), T10 = Dugan et al. (2005), T11 = Bova et al. (2006), T12 = Bova et al. (2012), T13 = Hillen et al. (2012), T14 = Hillen et al. (2013). 4-point scale rating: +++ = excellent, ++ = good, + = fair, 0 = poor, empty space = COSMIN rating not applicable. n/a = not applicable. (DOCX) [file pone.0106844.s002.docx]

# Appendix S3: Detailed results for the COSMIN checklist with 4-point scale rating

| *Box* | *COSMIN psychometric properties/Items** | *study IDs/Rating scores* | | | | | | | | | | | | | |
| --- | --- | --- | --- | --- | --- | --- | --- | --- | --- | --- | --- | --- | --- | --- | --- |
|  |  | **T1** | **T2** | **T3** | **T4** | **T5** | **T6** | **T7** | **T8** | **T9** | **T10** | **T11** | **T12** | **T13** | **T14** |
| **IRT** | **IRT** |  |  |  |  |  |  |  |  |  |  |  |  |  |  |
| **A** | **Internal consistency** | **T1** | **T2** | **T3** | **T4** | **T5** | **T6** | **T7** | **T8** | **T9** | **T10** | **T11** | **T12** | **T13** | **T14** |
| 1 | Does scale consist of effect indicators, i.e. is it based on a reflective model? | ok | ok | ok | ok | ok | ok | ok | ok | ok | ok | ok | ok | ok | ok |
| 2 | Percentage of missing items given? | ++ | ++ | ++ | +++ | ++ | ++ | +++ | ++ | ++ | ++ | ++ | ++ | ++ | ++ |
| 3 | Description of how missing items were handled? | + | + | +++ | + | + | + | + | +++ | + | ++ | +++ | +++ | +++ | + |
| 4 | Sample size included in internal consistency analysis adequate? | +++ | +++ | +++ | +++ | +++ | +++ | +++ | +++ | ++ | +++ | ++ | +++ | +++ | +++ |
| 5 | Unidimensionality of scale checked, i.e. factor analysis or IRT model applied? | 0 | 0 | +++ | +++ | 0 | +++ | +++ | +++ | +++ | +++ | +++ | +++ | +++ | +++ |
| 6 | Sample size included in unidimensionality analysis adequate? | 0 | 0 | +++ | +++ | 0 | +++ | +++ | +++ | +++ | +++ | ++ | +++ | +++ | +++ |
| 7 | Internal consistency statistic calculated for each scale separately? | +++ | +++ | +++ | +++ | +++ | +++ | +++ | +++ | +++ | +++ | +++ | +++ | +++ | +++ |
| 8 | Any important flaws in design or method of the study? | +++ | +++ | +++ | +++ | 0 | 0 | +++ | +++ | +++ | +++ | +++ | +++ | +++ | +++ |
| 9 | For CTT, continuous scores: Cronbach's alpha calculated? | +++ | +++ | +++ | +++ | +++ | +++ | +++ | +++ | +++ | +++ | +++ | +++ | +++ | +++ |
| 10 | For CTT, dichotomous scores: Cronbach's alpha or KR-20 calculated? | n/a | n/a | n/a | n/a | n/a | n/a | n/a | n/a | n/a | n/a | n/a | n/a | n/a | n/a |
| 11 | For IRT, goodness of fit statistic at global level calculated? | n/a | n/a | n/a | n/a | n/a | n/a | n/a | n/a | n/a | n/a | n/a | n/a | n/a | n/a |
|  | **Final score Box A** | **0** | **0** | **++** | **+** | **0** | **0** | **+** | **++** | **+** | **++** | **++** | **++** | **++** | **+** |
| **B** | **Reliability** |  | **T2** |  |  |  |  | **T7** |  | **T9** |  | **T11** |  | **T13** |  |
| 1 | Percentage of missing items given? |  | ++ |  |  |  |  | ++ |  | ++ |  | ++ |  | ++ |  |
| 2 | Description of how missing items were handled? |  | + |  |  |  |  | + |  | + |  | ++ |  | ++ |  |
| 3 | Sample size included in analysis adequate? |  | +++ |  |  |  |  | +++ |  | 0 |  | 0 |  | ++ |  |
| 4 | At least two measurements available? |  | +++ |  |  |  |  | +++ |  | +++ |  | +++ |  | +++ |  |
| 5 | Administrations independent? |  | ++ |  |  |  |  | ++ |  | + |  | ++ |  | +++ |  |
| 6 | Time interval stated? |  | +++ |  |  |  |  | +++ |  | +++ |  | +++ |  | +++ |  |
| 7 | Patients stable in the interim period on the construct to be measured? |  | + |  |  |  |  | + |  | 0 |  | ++ |  | ++ |  |
| 8 | Time interval appropriate? |  | +++ |  |  |  |  | 0 |  | 0 |  | +++ |  | + |  |
| 9 | Test conditions similar for both measurements? |  | ++ |  |  |  |  | ++ |  | ++ |  | +++ |  | ++ |  |
| 10 | Any important flaws in design or methods of the study? |  | +++ |  |  |  |  | +++ |  | +++ |  | +++ |  | +++ |  |
| 11 | For continuous scores: intraclass correlation coefficient (ICC) calculated? |  | ++ |  |  |  |  | + |  | + |  | ++ |  | + |  |
| 12 | For dichotomous/nominal/ordinal scores: kappa calculated? |  | n/a |  |  |  |  | n/a |  | n/a |  | n/a |  | n/a |  |
| 13 | For ordinal scores: weighted kappa calculated? |  | n/a |  |  |  |  | n/a |  | n/a |  | n/a |  | n/a |  |
| 14 | For ordinal scores: weighting scheme described? |  | n/a |  |  |  |  | n/a |  | n/a |  | n/a |  | n/a |  |
|  | **Final score Box B** |  | **+** |  |  |  |  | **0** |  | **0** |  | **0** |  | **+** |  |
| **C** | **Measurement error** |  |  |  |  |  |  |  |  |  |  |  |  |  |  |

… Continued on the next page

| **D** | **Content validity** | **T1** |  |  |  |  | **T6** | **T7** |  |  |  | **T11** |  | **T13** |  |
| --- | --- | --- | --- | --- | --- | --- | --- | --- | --- | --- | --- | --- | --- | --- | --- |
| 1 | Assessment of whether all items refer to relevant aspects of the construct? | +++ |  |  |  |  | +++ | +++ |  |  |  | +++ |  | +++ |  |
| 2 | Assessment of whether all items are relevant for the study population | +++ |  |  |  |  | 0 | +++ |  |  |  | +++ |  | +++ |  |
| 3 | Assessment of whether all items are relevant for the purpose of instrument | ++ |  |  |  |  | ++ | ++ |  |  |  | ++ |  | ++ |  |
| 4 | Assessment of whether all items together reflect the construct? | +++ |  |  |  |  | +++ | +++ |  |  |  | +++ |  | +++ |  |
| 5 | Any important flaws in design or method of the study? | +++ |  |  |  |  | + | +++ |  |  |  | +++ |  | +++ |  |
|  | **Final score Box D** | **++** |  |  |  |  | **0** | **++** |  |  |  | **++** |  | **++** |  |
| **E** | **Structural validity** |  |  | **T3** | **T4** |  | **T6** | **T7** | **T8** | **T9** | **T10** | **T11** | **T12** | **T13** | **T14** |
| 1 | Does scale consist of effect indicators, i.e. is it based on a reflective model? |  |  | ok | ok |  | ok | ok | ok | ok | ok | ok | ok | ok | ok |
| 2 | Percentage of missing items given? |  |  | ++ | +++ |  | ++ | +++ | ++ | ++ | ++ | ++ | ++ | ++ | ++ |
| 3 | Description of how missing items were handled? |  |  | +++ | + |  | + | + | +++ | + | ++ | +++ | +++ | +++ | + |
| 4 | Sample size included in analysis adequate? |  |  | +++ | +++ |  | +++ | +++ | +++ | ++ | +++ | ++ | +++ | +++ | +++ |
| 5 | Any important flaws in design or method of the study? |  |  | + | + |  | 0 | + | +++ | + | +++ | +++ | +++ | +++ | +++ |
| 6 | For CTT: exploratory or confirmatory factor analysis performed? |  |  | +++ | +++ |  | +++ | +++ | +++ | +++ | +++ | +++ | +++ | +++ | +++ |
| 7 | For IRT: tests for determining (uni-) dimensionality of the items performed? |  |  | n/a | n/a |  | n/a | n/a | n/a | n/a | n/a | n/a | n/a | n/a | n/a |
|  | **Final score Box E** |  |  | **+** | **+** |  | **0** | **+** | **++** | **+** | **++** | **++** | **++** | **++** | **+** |
| **F** | **Hypotheses testing** | **T1** | **T2** | **T3** | **T4** | **T5** | **T6** | **T7** | **T8** | **T9** | **T10** | **T11** | **T12** | **T13** | **T14** |
| 1 | Percentage of missing items given? | ++ | ++ | ++ | +++ | ++ | ++ | +++ | ++ | ++ | ++ | ++ | ++ | ++ | ++ |
| 2 | Description of how missing items were handled? | + | + | +++ | + | + | + | + | +++ | + | ++ | +++ | +++ | +++ | + |
| 3 | Sample size included in analysis adequate? | 0 | 0 | ++ | + | 0 | 0 | ++ | ++ | 0 | ++ | 0 | + | +++ | ++ |
| 4 | Hypotheses regarding correlations or mean differences formulated a priori? | + | ++ | ++ | ++ | 0 | + | + | +++ | + | + | + | ++ | ++ | ++ |
| 5 | Expected direction of correlations or mean differences included in hypotheses? | ++ | +++ | +++ | ++ | ++ | ++ | ++ | ++ | ++ | ++ | ++ | ++ | +++ | +++ |
| 6 | Expected absolute/relative magnitude of correlations/mean differences included in hypotheses? | ++ | ++ | ++ | +++ | ++ | ++ | ++ | ++ | ++ | ++ | ++ | ++ | +++ | +++ |
| 7 | For convergent validity: adequate description provided of comparator instrument(s)? | +++ | +++ | +++ | +++ | 0 | +++ | +++ | +++ | +++ | +++ | +++ | +++ | +++ | +++ |
| 8 | For convergent validity: measurement properties of comparator instrument(s) adequately described? | 0 | 0 | + | 0 | 0 | 0 | 0 | + | 0 | + | + | +++ | 0 | 0 |
| 9 | Any important flaws in design or method of the study? | +++ | 0 | +++ | +++ | 0 | 0 | +++ | +++ | +++ | + | +++ | +++ | +++ | +++ |
| 10 | Design and statistical methods adequate for hypotheses to be tested? | +++ | ++ | ++ | +++ | + | ++ | +++ | +++ | +++ | +++ | ++ | ++ | +++ | +++ |
|  | **Final score Box F** | **0** | **0** | **+** | **0** | **0** | **0** | **0** | **+** | **0** | **+** | **0** | **+** | **0** | **0** |

… Continued on the next page

| **G** | **Cross-cultural validity** |  |  |  | **T4** | **T5** |  |  | **T8** |  |  |  |  |  | **T14** |
| --- | --- | --- | --- | --- | --- | --- | --- | --- | --- | --- | --- | --- | --- | --- | --- |
| 1 | Percentage of missing items given? |  |  |  | +++ | ++ |  |  | ++ |  |  |  |  |  | ++ |
| 2 | Description of how missing items were handled? |  |  |  | + | + |  |  | +++ |  |  |  |  |  | + |
| 3 | Sample size included in analysis adequate? |  |  |  | +++ | +++ |  |  | +++ |  |  |  |  |  | +++ |
| 4 | Both the original language in which instrument was developed and language in which instrument was translated described? |  |  |  | +++ | 0 |  |  | +++ |  |  |  |  |  | +++ |
| 5 | Expertise of people involved in translation process adequately described? |  |  |  | ++ | + |  |  | + |  |  |  |  |  | + |
| 6 | Did translators work independently from each other? |  |  |  | + | + |  |  | + |  |  |  |  |  | + |
| 7 | Items translated forward and backward? |  |  |  | + | 0 |  |  | + |  |  |  |  |  | + |
| 8 | Adequate description of how differences between the original and translated versions were resolved? |  |  |  | ++ | ++ |  |  | ++ |  |  |  |  |  | ++ |
| 9 | Translation reviewed by a committee? |  |  |  | ++ | ++ |  |  | ++ |  |  |  |  |  | ++ |
| 10 | Instrument pre-tested (e.g. cognitive interviews) to check interpretation, cultural relevance of the translation, and ease of comprehension? |  |  |  | +++ | 0 |  |  | ++ |  |  |  |  |  | 0 |
| 11 | Sample used in the pre-test adequately described? |  |  |  | + | + |  |  | + |  |  |  |  |  | + |
| 12 | Samples similar for all characteristics except language and/or cultural background? |  |  |  | 0 | 0 |  |  | + |  |  |  |  |  | + |
| 13 | Any important flaws in design or methods of the study? |  |  |  | +++ | 0 |  |  | +++ |  |  |  |  |  | +++ |
| 14 | For CTT: confirmatory factor analysis performed? |  |  |  | 0 | 0 |  |  | 0 |  |  |  |  |  | +++ |
| 15 | For IRT: differential item function (DIF) between language groups assessed? |  |  |  | n/a | n/a |  |  | n/a |  |  |  |  |  | n/a |
|  | **Final score Box G** |  |  |  | **0** | **0** |  |  | **0** |  |  |  |  |  | **0** |
| **H** | **Criterion validity** |  |  |  |  |  |  |  |  |  |  |  |  |  |  |
| **I** | **Responsiveness** |  |  |  |  |  |  |  |  |  |  |  |  |  |  |

* Description of item content altered to fit this table. For exact item content see COSMIN website (www.cosmin.nl). Study IDs: T1 = Anderson & Dedrick (1990), T2 = Thom et al. (1999), T3 = Freburger et al. (2003), T4 = Glattacker et al. (2007), T5 = Krajewska-Kulak et al. (2011), T6 = Leisen & Hyman (2001), T7 = Hall et al. (2002), T8 = Bachinger et al. (2008), T9 = Donnelly et al. (2011), T10 = Dugan et al. (2005), T11 = Bova et al. (2006), T12 = Bova et al. (2012), T13 = Hillen et al. (2012), T14 = Hillen et al. (2013). 4-point scale rating: +++ = excellent, ++ = good, + = fair, 0 = poor, empty space = COSMIN rating not applicable. n/a = not applicable.
